# Building a healthcare data warehouse: considerations, opportunities, and challenges

**DOI:** 10.3389/fdgth.2025.1691142

**Published:** 2025-12-16

**Authors:** Tamara Knezevic Ivanovski, Sailish Honap, Rade Matic, Srdjan Markovic, Laurent Peyrin-Biroulet

**Affiliations:** 1 Department of Gastroenterology and Hepatology, University City Hospital Zvezdara, Belgrade, Serbia; 2 Faculty of Medicine, University of Belgrade, Belgrade, Serbia; 3 Department of Gastroenterology, St George’s University Hospitals NHS Foundation Trust, London, United Kingdom; 4 School of Immunology and Microbial Sciences, King’s College London, London, United Kingdom; 5 Department for Information Systems and Technologies, Belgrade Academy for Business and Arts Applied Studies, Belgrade, Serbia; 6 Department of Gastroenterology, CHRU Nancy, Inserm NGERE, Lorraine University, Vandoeuvre, France

**Keywords:** data warehourse, inflammatory bowel disease, ETL/ELT, data integration and interoperability, artificial intelligence in healthcare

## Abstract

The increasing digitalization of health systems is accelerating the transition towards a new era of data-driven, evidence-based care. This has profound implications for clinical practice, performance evaluation, policy making and biomedical research. At the heart of this transformation lies a healthcare data warehouse (DW), which functions as a critical infrastructure for aggregating, standardizing, and analyzing diverse clinical and administrative data. When well-designed and implemented, DWs provide clinicians with timely access to comprehensive, longitudinal patient data, enabling more informed decision-making, enhancing care quality, and improving outcomes. For researchers, these repositories offer opportunities for population-level analytics, predictive modeling, and large-scale health service research, enabling insights into disease patterns, healthcare utilization, and system inefficiencies. Centralizing clinical and administrative data in a DW allows for more frequent, nuanced analyses, increasing the precision and responsiveness of care. However, developing an effective DW requires careful consideration of system architecture, data governance, and interoperability. These foundational components support the robust ETL/ELT frameworks that ensure data quality, consistency, and readiness for analysis across diverse and evolving data streams. Beyond supporting individual patient care, DWs act as essential drivers of scalable research, operational efficiency, and evidence-based health policy. Their successful implementation marks a pivotal step toward achieving personalized, high-quality, and cost-effective healthcare in the digital transformation age. This paper reviews the existing literature to outline the process of building and implementing a data warehouse, introducing real-world disease-specific applications. BiotherDW connects theoretical frameworks with practical healthcare applications by demonstrating how traditional data warehouse design can be adapted for national-scale digital health infrastructures.

## 1 Introduction

The digitalization of healthcare systems has significant scope for improving healthcare delivery and patient outcomes (1–3), as it moves towards evidence-based, data-led health. Much of this change has been attributed to the widespread adoption of Electronic Health Records (EHRs), which facilitate the entry, processing, storage, and retrieval of digital health data (4). This, in turn, can support vast quantities of data such as clinical practice, healthcare monitoring and evaluation, policymaking, and clinical research (5, 6). Modern healthcare systems produce enormous amounts of data through EHRs. They are built to manage information at the individual patient level, including clinical notes, laboratory results, imaging, prescriptions, procedures, and diagnoses. These contain admission-discharge-transfer records, billing, scheduling, claims processing,  human resources, and payroll systems (7, 8). Building on this solid foundation in digital transformation, it is important to evaluate how these systems support evidence-based, data-led healthcare and the challenges encountered when scaling up to enterprise-level data management (9). However, the transition from isolated data silos to integrated, enterprise-level data environments presents new challenges—particularly regarding standardization, interoperability, data modeling, advanced analytics, and the reuse of meaningful data. This highlights the need for centralized repositories that can integrate these diverse data sources (10, 11).

Clinical data warehouses (DWs) are crucial as they are specifically designed for integrating and standardizing information from a variety of sources, including EHR data and administrative databases (12). Through this transformation process, fragmented data becomes centralized and analyzable, helping with healthcare decisions and encouraging teamwork among healthcare professionals (13). The rise of clinical DWs as a mature and widely accepted solution for integrating various healthcare data has provided support for decision-making, enhancing it through research, development, and improved measurement quality (2). This paper aims to integrate traditional architectural frameworks on DW with modern solutions, such as data lakes and lakehouses, analytics based in AI, and data privacy. It also presents several real-world with the goal to help stakeholders be more prepared for the ever-changing landscape of digital health data infrastructure.

DW stores data in a structured form, making it more accessible for analysis. Thus, the DW serves as the backbone of data-driven decision-making across both clinical and organizational domains (14). However, alternative architectures, such as data lakes and data lakehouses, have emerged to address the growing volume of health data (15). To manage data lakes properly, organizations are incorporating data lakehouses, which enhance lake environments with warehouse-level governance and transactional consistency (16). In each of these alternative architectures, there is an inherent trade-off between governance, scalability, and analytical performance parameters (6). A focus on data warehousing provides a grounded and evidence-based perspective on what currently works in healthcare data management and what needs to change to make the next generation of data-driven medicine a reality (17).

## 2 Methods

This narrative review sought to provide an overview of the existing literature on building health care data warehouses. A comprehensive search was conducted in MEDLINE (via PubMed) of articles published from 2000 to 2025. Keywords related to data warehousing and information technology in health, such as “data warehouse”, “health-care data infrastructure”, “interoperability”, and “electronic health records”, as well as “clinical data” were employed and combined in various ways to find relevant literature. No strict set of pre-defined criteria for article inclusion was utilized for this narrative review—selection was based on the relevance and its originality/contribution towards the field. Additional references were identified through hand searching of reference lists to identify relevant papers.

## 3 Governance and analytics in healthcare: data architecture patterns

In the context of modern healthcare analytics, there were previously three basic data architecture patterns: DW, data lake, and data lakehouse, supporting business intelligence (BI) and data-driven decision-making (14, 18). Each fulfills a different need an organization has in storing, structuring, and analyzing information, providing a spectrum of uses from standardized reports through exploratory data science projects to AI applications for encoding information (19).

A DW is a tightly governed, centralized repository that brings together detailed data from multiple sources (1). It follows the schema-on-write paradigm, so data is validated and organized before being loaded–usually into star or snowflake schemas to improve report processing speed (20). This approach ensures high data fidelity and supports repeatable, trustworthy analytics so DWs are especially good at making trusted operational reports, regulatory filings and performance metrics for the hospital (19, 20).

Consequently, a data lake is a vast, free-form repository capable of holding raw and partly structured data in its original state (21). With a focus on a schema-on-read pattern, data lakes enable you to take in wide-ranging sets of data types, including structured (like EHR exports), semi-structured (like JSON logs), and unstructured formats (cell notes from patients to their caregivers) (21, 22). This architecture supports real-time data ingestion, large-scale exploration, and data lakehouse pipelines (14, 21). However, it frequently requires supplementary metadata and governance frameworks to ensure data quality and make it discoverable (22). Nevertheless, the lack of angular control and vigorous governance in data lakes often leads to poor data discovery, different quality, and regulatory hazards, making them inappropriate for clinical decision support, where people must have stabilized tracing information that has been verified (23).

To bridge this gap, the data lakehouse architecture proposes a new pattern where flexibility and scalability coexist with integrity and metadata governance in DW, making possible not only transparent analytics across unstructured and structured health data but also active interrogation (24). It promotes the integration of business intelligence, AI, advanced analytics and other types of data. Lakehouses encourage innovation while maintaining the necessary auditability for application data by allowing concurrent access to raw and refined data sets.

Altogether, these architectures are not distinct, but increasingly interoperable, forming layered data ecosystems that correspond with real-world data capture and demand for different types of analytics, from operational intelligence to predictive modeling (25). Data lakes support exploration, lakehouses enable innovation, and DWs remain the cornerstone of consistent reporting and governance (26). Organizations typically adopt a DW when their main objective is to ensure reliable, standardized business reporting supported by robust governance and regulatory compliance. DW means stable, curated data models that are easy to maintain and integrate with BI tools such as Power BI or Tableau (19, 26). With the potential for better performance on analytical queries, team adoption is easier because it is SQL, and there is greater data accuracy. This is vital where precision and reliability take precedence over flexibility in many industries (2, 26). The choice of DW as the principal data management system for healthcare organizations is prompted by the stringent requirements of domains on data quality, semantic consistency, and regulatory compliance (27). By enforcing schemas and metadata, DW converts diverse clinical and administrative sources into coherent, high-integrity datasets, ensuring temporal consistency that schema-on-read data lakes cannot reliably provide (28, 29). They also offer mature SQL/BI tooling and auditable ETL/ELT and lineage mechanisms, ensuring traceable access to patient records at any point in time, supporting accountability under regulations like the Health Insurance Portability and Accountability Act (HIPAA) and the General Data Protection Regulation (GDPR) (6, 30). Lakes and lakehouses are scalable, but they fail to address governance and interoperability, which remains a challenge in the era of multisource healthcare (31). DWs that are well-governed in practice not only promote analytical adoption and stewardship but also imbue trust, so the advantages of conducting longitudinal, comprehensive analysis or follow-up across time periods are enjoyed by many providers. This is why they remain as one of the primary foundations for high-integrity analytics (29, 32).

Nevertheless, most healthcare organizations still rely on well-organized DW as the foundation for their analytical infrastructure, using data lakes and lakehouses primarily as auxiliary environments for large-scale learning and data exploration (22, 26). From a strategy perspective, the DW remains the most trusted layer for ensuring accuracy, lineage auditing, and alignment with clinical facts. Newer architectures are gradually adopting these foundational characteristics, reflecting a shift toward a more standardized, “factory assembly line” approach to data management (6). So, while data lakes and lakehouses enable analyses beyond clinical applications, they do not replace the foundational role of DW in healthcare systems. They extend it instead into today's hybrid world, where map-and-reduce clusters, deterministically reworking cloud architectures, historical record management, and analytics meet modern scientific workflows and big-data environments (23, 24).

## 4 Core architectural models for healthcare data integration

Based on their data integration strategies and governance models, healthcare DWs and integrated data repositories (IDRs) can be divided into four main kinds of architecture (32, 33). The general model of architecture is the traditional and most common, where independent clinical and research stores are built into a centralized staging layer. This handles extraction, transformations, and harmonization. This design provides strong control over data quality and consistency, supporting large-scale medical data mining (30). Based on this, the biobank-specific architecture is tailored for managing blood specimens and gene data through a centralized bio-sample database that connects biological materials to their associated clinical metadata (5). In contrast, the application-layer or user-controlled architecture eliminates the need for a persistent staging layer, instead performing data preprocessing and integration dynamically at the time of query execution, providing flexibility for exploratory and ad-hoc research (34). The federated architecture model enables data from different institutions to be distributed countrywide, with real-time virtual integration through adapters and regular preprocessing rules. This model is particularly suited to multi-institutional collaborations, such as national or international research networks, where secure data sharing is essential (35).

Collectively, these architectural approaches outline the range between centrally and decentrally integrated systems, each presenting different trade-offs in scalability, interoperability, and governance within healthcare data warehousing environments.

## 5 Data research networks and data registries

Healthcare data ecosystems increasingly combine institutional data architectures, such as DW, data lakes, and lakehouses, with collaborative infrastructures like disease-specific registries and data research networks (DRNs) (14, 36). Each provides distinct advantages and limitations, and together they address the challenges of fragmentation, scalability, and accessibility (22). DRNs are federated infrastructures that connect multiple healthcare organizations to enable large-scale, collaborative research while maintaining local data control and patient privacy. They rely on standard data models such as OMOP or PCORnet to standardize data across institutions, allowing reproducible and comparable analyses (37). These models provide a *virtual centralization* of data while preserving local control, handy for multi-institutional studies where data-sharing restrictions apply (18). Disease-specific registries, in contrast, are focused databases that systematically collect and manage detailed information on patients with a particular condition, procedure, or treatment within a defined clinical area (e.g., inflammatory bowel disease, oncology, or rare diseases) (7, 15, 38, 39). These registries support clinical research, quality improvement, and epidemiological monitoring by providing curated, high-quality datasets derived from institutional DWs. Together, DRNs and disease-specific registries represent the collaborative layer of modern healthcare data architecture—extending institutional DWs toward population-level and cross-institutional insights (38).

At the organizational level, a DW integrates clinical, administrative, and laboratory data into standardized, high-quality datasets that often serve as the primary source for disease-specific registries (27). These registries, in turn, feed into multi-institutional DRNs that harmonize local data models through frameworks such as OMOP or PCORnet, enabling federated analytics and large-scale clinical studies (40). Meanwhile, data lakes and lakehouses extend this architecture by supporting multimodal data types imaging, genomics, sensor data, allowing registries and DRNs to incorporate richer, unstructured information for advanced analytics and machine learning (24). Together, these layers form a complementary data architecture: institutional DWs provide governance and quality, registries ensure clinical specificity, and DRNs enable collaborative, large-scale research.

DRN, data registries, along with DW, create a complementary ecosystem that integrates diverse data streams into cohesive, high-quality, and actionable insights. This integration facilitates improved clinical decision-making, enhances the efficiency of health systems, and supports informed health policy development on a large scale. However, their true value in interface applications can only be judged by how well they serve their diverse stakeholders, who then turn to consider the perspectives of patients, doctors, administrators, managers, researchers, and politicians.

## 6 Stakeholders in healthcare data: who benefits and how?

**Figure 1:**
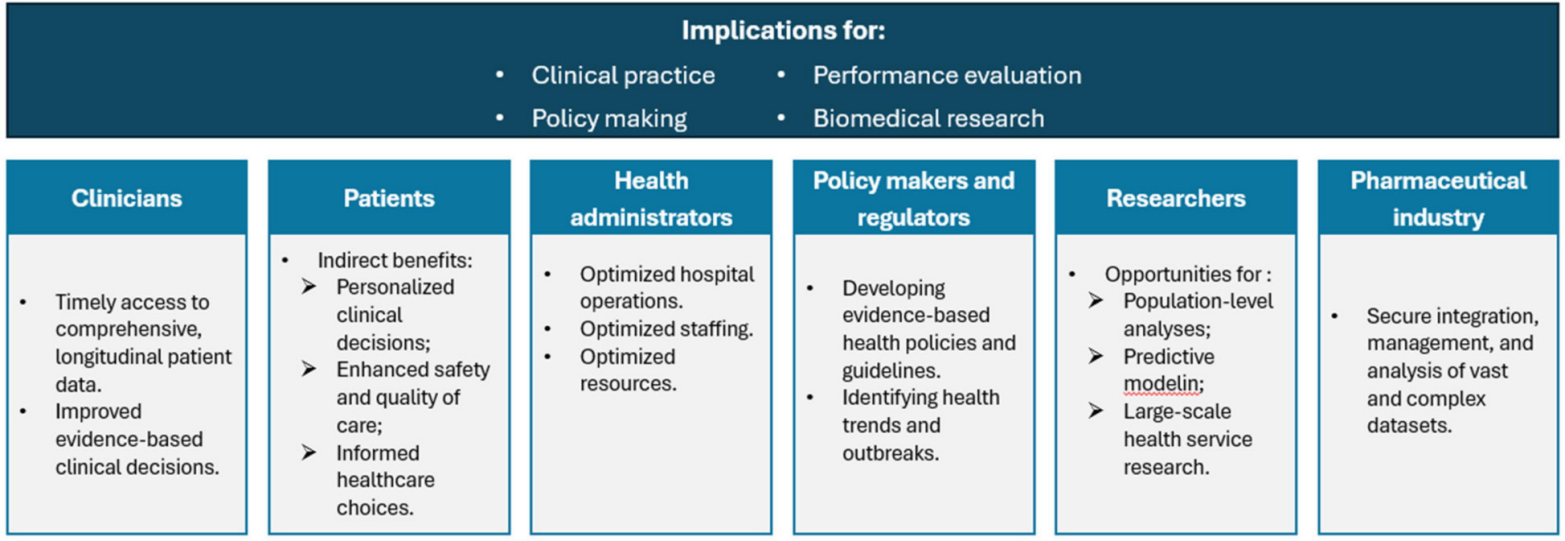
Stakeholders in healthcare data: who benefits and how?

Healthcare data from DWs serves a variety of stakeholders, including patients and service users who depend on information to make informed choices for their health and care (22). Patients and service users rely on these data to make informed choices about their health and care (Figure 1). By providing clinicians with access to each patient's comprehensive medical history, DWs facilitate more personalized and effective care (11, 25).

For clinicians, it provides a complete view of and seamless access to each patient record with relevant patient history, laboratory investigations, and outcome data, leading to improved evidence-based clinical decisions and patient outcomes. However, clinicians' need for up-to-date, real-time data can conflict with other priorities: for example, researchers often require stable, static datasets to conduct rigorous analyses. Balancing these operational needs with research requirements is a key challenge in DW governance.

Health administrators and managers use DWs for operational insights to optimize hospital operations, staffing, and resources. This focus on control can sometimes put administrators at odds with academic stakeholders: administrators may favor strict oversight, whereas researchers advocate for broad data access to drive innovation (41, 42).

Researchers and analysts use robust, integrated datasets from DWs to conduct population-level analyses, derive insights on disease patterns, treatment effectiveness, and patient outcomes, and support innovation through rapid access to standardized data (18, 23, 43). Rapid, standardized data accelerates research and innovation. However, research goals can also conflict with operational constraints due to the requirement for extra curation or approval, potentially delaying analysis (18, 39).

Policy makers and regulators rely on DWs for developing evidence-based health policies and guidelines, effectively managing public health by identifying health trends and outbreaks and increasing transparency and accountability through detailed performance assessments. By identifying trends and outbreaks, DWs enable more proactive, evidence-driven policies. Nevertheless, in urgent situations, policy demands can push for rapid data sharing in ways that challenge standard governance or privacy safeguards (5, 18, 38).

Patients benefit indirectly through more personalized clinical decisions, enhanced safety and quality of care due to systematic monitoring, and empowerment to make informed healthcare choices (19). The long-term viability of digital health ecosystems hinges on sustaining patient trust. Ensuring transparency, accountability, and demonstrable value to patients is therefore essential to maintaining the social license for data-driven innovation.

A robustly designed DW represents a critical asset in the pharmaceutical industry, providing a secure and unified platform for integrating, managing, and analyzing large, complex datasets (39). Through its standardized and centralized structure, it facilitates regulatory compliance as an essential requirement in this tightly controlled domain (44). However, the substantial commercial value of such data necessitates transparent oversight and stringent ethical governance.

Further ethical and governance challenges: In addition to tensions specific to stakeholder interests, there are even more important political and moral issues that must be addressed. Questions of data ownership remain unresolved: institutions typically claim ownership of health records, but patients are increasingly asserting their rights over their own data (33). Explicit policies that respect patient rights and promote use of data for the public good should regulate DWs. Moreover, a whole series of questions about the political, moral, and technical aspects new technology has brought to light now need our attention. A DW must be operated with clear policies that safeguard patient rights and ensure data are used for the common good.

## 7 Challenges in healthcare DW

By systematically outlining challenges bridging data integration, quality, security, and regulatory compliance, this review aims to offer a comprehensive perspective for practitioners, researchers, and policymakers. It will not only clarify existing complexities but also establish a foundation for future research and advancement in healthcare DW systems.

Despite their transformative potential, the quality and reliability of the information in healthcare DWs can be severely compromised by issues encountered during their implementation and operation. Inadequate management of these challenges can result in unreliable data insights, which may threaten patient safety and reduce the effectiveness of clinical interventions. Healthcare organizations require scalable, high-performance, and secure infrastructures capable of handling rapidly expanding datasets while maintaining data integrity and accessibility (45).

Healthcare systems operate under stringent regulatory and ethical constraints that shape data governance, privacy, and security. Accordingly, strategies for ensuring patient confidentiality and ethical data use directly influence the integral DW architecture and its operational framework (46).

### 7.1 Data integration complexity

One of the major challenges in healthcare data warehousing is how to integrate diverse data sources. Healthcare data comes from many sources, including hospitals, clinics, laboratories, and government health agencies, each with its own data formats and standards (47). The variety includes EHRs, lab results, imaging data, billing records, and more, creating inherent complexity when trying to combine this data into a single DW (48). Adding to the difficulty, there are many data types beyond structured information (like coded diagnoses and lab results) and unstructured data (such as clinical notes or x-ray images), which require different processing and storage approaches (49). Managing it in this way ensures that whatever information is received is handled with care so as to be accessible and grasped by people. In addition, the use of different terminologies and standards, such as ICD codes, SNOMED CT, or any local coding scheme, easily makes interoperability difficult to achieve effectively or is responsible for semantic inconsistency (48). Aligning these terminologies demands careful mapping and normalization efforts to support accurate cross-institutional data queries and analytics.

In addition to technical heterogeneity, the biggest obstacle is integrating organizational and infrastructure-related issues (27, 29). This often causes problems when old hospital automation systems need to interface with modern DWs, as well as when clinical information systems try to operate within newer ones (47). Complementary mismatches between legacy platforms and hardware can also pose barriers. These platforms often lack open structures or freely available APIs for information exchange between systems, so data does not flow as freely as users would like (39). Additionally, a lack of communication and collaboration between IT personnel and clinical staff compromises successful requirements gathering and system design. In the worst-case scenario, these issues lead to suboptimal data models that lower the warehouse's utilization factor below expected levels (50). Maintaining accurate data provenance and audit trails presents additional complexity, as healthcare systems must document data origins and transformations rigorously to ensure reliability and compliance with legal standards. Maintaining robust provenance and audit trails adds operational complexity, as healthcare systems must rigorously document data sources and transformation steps to safeguard reliability and meet regulatory requirements (51).

### 7.2 Data quality and consistency

Organization and completeness of data presentation posed challenges in this paper. Meanwhile, accuracy is always crucial for successfully rolling out or disseminating data products that truly reach a broad audience. Therefore, both quality and data sources need improvement. For reliable healthcare analysis, high-quality data is essential. Healthcare DWs often aggregate data from multiple institutions, making the preservation of precise, byte-level data integrity a formidable challenge (52). It may later become impossible to determine how much of the original source material was lost due to copy-and-paste operations (another form of substitution error). Various data collection methods, human errors during encoding, and missing, null, “don't know”, or “refuse to answer” values threaten to make integrated data sets either incomplete or inaccurate or both. Poor-quality data can carry through the entire analytical process, directly impacting patient outcomes or the validity of decision support systems (53). Nowadays, mitigation strategies increasingly rely on combined verification and data cleaning techniques, AI-assisted anomaly detection, deduplication, and normalization (54). These automated systems keep data clean by continuously reporting anomalies they detect to system administrators, eliminating the need for manual intervention while increasing both trust and efficiency.

The next issue concerning data quality involves bias and imbalances in datasets. EHR and other healthcare data banks can reflect demographic, socioeconomic, or clinical biases embedded in healthcare provision and diagnosis documents (53). Still, many areas underrepresent minorities, which can alter data distribution. Failing to address this can lead to unfairness and errors in AI or ML models trained on such databases (54). Reducing bias requires systems for ongoing algorithm monitoring and model recalibration to uphold fairness. Without these safeguards, models may propagate inequities. Approaches such as bias-aware resampling and transparent evaluation help detect and mitigate underlying data imbalances (55).

In modern healthcare, real-time data integration is increasingly regarded as a crucial tool for rapid intervention. However, the challenge of maintaining data freshness and synchronization continues to be a technological obstacle for healthcare DWs (56). Real-time integration of lab results along with clinical monitoring data disrupts the flow, due to both speed and scale (57). To manage high-velocity data, it is vital to design streaming infrastructures that can handle scalable ingestion and processing. Additionally, it is critically important to keep consistency and cohesion between real-time and batch datasets to ensure accurate clinical decision-making (47). This environment requires a robust streaming architecture capable of handling high data input rates. These challenges necessitate scalable infrastructure and advanced ETL/ELT (Extract, Transform, Load/ Extract, Load, Transform) pipelines specifically designed for the dynamic nature of medical data.

### 7.3 Security and privacy concerns

Since healthcare data is sensitive, strong data protection is a top priority. Managing patient consent dynamically within health environments is essential for fulfilling legal responsibilities that benefit patients (46). Traditional static consent methods are often insufficient in complex data ecosystems supporting multiple secondary uses. Systems must incorporate transparency and accountability, allowing patients to decide who can access their data and for what purposes (46).

The cornerstones of protecting privacy in healthcare DWs are data encryption and robust access control. There is a growing emphasis on models of attribute-based access control (ABAC) nowadays, which can adaptively define security policies in more detail and include various attributes related to context, rather than just relying on set user roles (30). Protocol-oriented XPOTAM techniques protect valuable information during data sharing, cloud migration, and similar processes (58). The same is true for tokenization with synthetic data. However, these measures not only prevent sensitive data from being disclosed without authorization but also help organizations meet strict regulatory requirements such as the GDPR and HIPAA. Implementing these controls requires technological solutions along with organizational processes that align with the regulatory standards of the respective field (30).

But despite the advances, cloud-based DWs remain vulnerable to cybersecurity threats like data breaches or ransomware (59). In a distributed data architecture, as data moves across multiple systems and networks, it increases the attack surface unless strong protective measures are in place. An ongoing operational challenge is balancing the needs of clinical users and researchers for easy access with strict security controls (46). Effective risk management frameworks and continuous security monitoring are vital for protecting sensitive patient data in this rapidly changing landscape.

### 7.4 Scalability and performance issues

Healthcare DWs need to load information from a wide variety of sources, such as population health records, images, genetic information and wearables. Big data poses significant challenges for both storage and processing infrastructures, so solutions must be scalable. NoSQL databases outperform traditional SQL relational systems for querying clinical data. This provides a significant advantage in terms of both performance and flexibility (45). To improve operational efficiency, a business needs to grow, and many are moving to cloud native architectures with elastic resource allocation, distributed processing and advanced data storage solutions. These are not only faster and more scalable architectures but also lower the price of entry, which is particularly important if cost is a consideration for project (60).

There is no doubt that limited resources for medical care in emerging countries present a challenge for both healthcare providers and those seeking care. Broken EHR systems, inconsistent patient IDs, and network issues hinder the integration and scalability of healthcare information. This means that during critical times, such as SARS-like outbreaks, we lack real-time capabilities for data processing and disease surveillance (45). To address these issues, researchers offer various solutions, including data marts designed for targeted analytics, secure ingestion pipelines with code generated and automatically adapted to meet local standards, and a lightweight architecture that requires minimal infrastructure (45).

Optimization strategies for healthcare DWs include employing dimensional modeling during cloud migration to streamline schema design, enhance query efficiency, and increase reporting flexibility (58). Automated ETL/ELT tools minimize manual intervention, enhance process consistency, and shorten deployment cycles (52). In distributed environments, advanced load balancing and provisioning techniques further improve system uptime. Testing with highly successful large-scale deployments demonstrates that the same technical environment can be transformed into something much more responsive than is typical for today's software and hardware combined.

### 7.5 Resource constraints and technical expertise

Building an effective healthcare DW depends on expertise in data science, medical informatics, and technology management. Reliance on highly specialized personnel can hinder scalability and delay the adaptation of DWs to evolving needs (52). Closing this technical gap requires programs for multi-skills education and capacity-building strategies that enhance both technical capabilities and relevant field knowledge. Complex healthcare DWs often need periodic maintenance to incorporate new data sources, update schemas, and refine procedures (52). However, integrating automated workflows with legacy systems to boost efficiency increases operational demands, requiring precise version control and mutual adaptability management. Additionally, financial constraints are likely to limit investment in advanced infrastructure and software, threatening the sustainability and growth of the warehouse (61).

An integrated, high-capacity data center defines the core of the precinct. This requires finding a way to bridge the cultural and communication gap between disciplines, so that people working in areas such as clinical patient care, technical support services, and administration can understand themselves not only from their own perspective but also from others, ultimately from diverse backgrounds (50). The extensive involvement of firm stakeholders makes it easier to understand expectations, builds more trust in working relationships, and enhances data stewardship. Interdisciplinary project management frameworks help promote shared accountability within stronger, more agile, and responsive healthcare environments (51).

### 7.6 Regulatory compliance and ethical issues

Healthcare DW must navigate to remain compliant, as data sharing procedures, security mechanisms, and AI governance frameworks constantly require assessment (62). Additionally, certain standards require that activities be pre-certified and auditable, which drives home once more the importance of complete documentation and quality management systems designed specifically for healthcare environments. This is particularly true in the case of healthcare data (46). Ethical considerations are also crucial in integrating AI into healthcare decision-making systems. Eliminating bias, ensuring transparency, and fairness when using AI models are all necessary prerequisites for medical staff to win patients' trust and deliver fair medical care (63). Regarding individual preferences, maintaining patient autonomy involves respecting informed consent and patients' wishes (46). Finding ways to balance innovation with privacy rights remains a complex challenge.

### 7.7 Interoperability and standardization

However, challenges arise during the mapping of local health care terminologies to these standards because there are so many linguistic varieties underpinning different places and the practices there vary over time, while documentation consistency is lacking (48). Data quality is affected by these challenges and so the analysis itself is less useful for the development of interoperable information systems (47). For example, with integrating various health IT systems, especially legacy platforms, it takes advanced middleware solutions and APIs to realize real-time data interchange between hospitals, laboratories, and repositories. Both technical and organizational coordination are required when integrating these efforts to cope with the differences between systems, update cycles, and governance silos (46). Middleware architecture ensures applications can interoperate and still specify what values to be taken for functions.

### 7.8 Managing evolution and digital transformation

Health care organizations are moving preferences from traditional on-site DWs to cloud native and hybrid architectures in order to harness the scalability, cost-effectiveness and improved analytic capabilities of these new kinds of systems (60). Data migration projects mean risk of losing data, system downtime, and performance deterioration. To avoid these risks, careful planning must be combined with rigorous validation procedures, including methods for ensuring continuity assurance (61). Post-migration optimization is necessary to ensure performance levels meet clinical and operational needs. AI-driven ETL/ELT processes and analytics functions are increasingly embedded inside healthcare DWs, supporting real-time analytics, anomaly detection, and predictive modeling (60). Building DWs that last into the future means creating flexible, modular infrastructures capable of integrating new analysis tools as they arrive (58). Automation tools that streamline ETL/ELT as well as data quality monitoring reduce manual labor, accelerate deployment, and provide greater consistency (52). Automated validation frameworks can improve data integrity, reducing pre-processing required by researchers and allowing for more rapid clinical insights (51). Also, business intelligence platforms have been demonstrated to improve decision-making and operational work within laboratory workflows (57).

The various stakeholders set the strategic goals of a DW. Delivering on these aims requires robust technical support. Further, we will outline practical steps for integrating and standardizing data across systems in healthcare settings.

## 8 How to construct a modern DW: key points

A modern DW must start with clear business goals and well-defined use cases, such as clinical research, operational reports, or strategic analytics. The key in this phase is identifying main stakeholders and end users and understanding their needs: analysts, clinicians, executives, and administrators all have different requirements, so they help guide the design and operation of your DW (39). Next, is to find all data sources and data integration pipeline links to ensure that incoming data is complete and supports interoperability. Also, consider regulatory requirements early on, such as HIPAA or GDPR, to ensure security and compliance. This builds trust in the DW for decision-making and prepares for future use cases involving new data types that may emerge but will need analysis (30). A modern DW consolidates large volumes of historical data from diverse sources into a centralized, cost-effective repository structured for business intelligence and advanced analytics (64–66). The models for modern DW architecture use cloud computing techniques, such as elastic computing and storage, partitioning data away from the processor itself, providing organizations with flexibility and greater operational efficiency (17, 67). The shift is driven mainly by the dramatic increase in data volumes and the resultant diversity of data sources, collectively known as big data, which necessitates scalable systems that can handle high-velocity and varied data formats (60). It is crucial to recognize the importance of modern DW projects, as they are indispensable for feeding advanced analytics systems that integrate AI/ML. Consisting in predictive insights complementary to traditional reporting (49, 68).

### 8.1 Architectural frameworks

The architecture of DW has changed from a heavy, unbalanced on-premises design to a flexible and scalable cloud architecture. On-premises DW, based on fixed hardware resources, flexible expansion, and fixed operating costs, inherently has limitations. In contrast, cloud-based DW platforms have elastic performance and cost-efficiency at a massive scale. Furthermore, their storage capability is almost limitless. Using cloud infrastructure, an organization can dynamically allocate system resources, swelling as demand rises and sheltering in times of decline. Thus, handling cost management effectively is possible (67, 69).

Modern DW design rests on a layered approach, with separate components for the ingestion, storage, processing, and analysis of data. Layers of data ingestion handle the extraction and loading from a wide range of heterogeneous sources and often integrate batch as well as streaming processing capabilities. The storage layer needs to absorb low-cost, large-scale retention of both structured and semi-structured data (67). It performs data transformations, aggregations, and query execution. Such layered processing engines often take advantage of vectorized processing and just-in-time compilation for superior performance. A basic concept adopted by modern architecture is to separate storage from computing resources and new resources to be utilized independently at higher efficiency, particularly in cloud environments (24). Additionally, by integrating with particular layers of AI/ML, this analysis procedure can have increased added value. Companies can integrate ML pipelines directly into the DBMS environment to support feature engineering and model production. Lakehouse, an emerging architectural paradigm, demonstrates the adaptability of today's data infrastructure for modern companies. After all, the human factor also counts for a great deal. But these new models will indeed mean great technical complexities as well as whole new ball games in organizational change management issues, for example with necessity for automatic governance systems and strategies spanning all areas (70).

### 8.2 DW architecture

For each integration and governance need, organizations may take upon themself one of a number of different DW architectures. The Inmon architecture stresses a model of normalized data across the whole enterprise, which ensures integrity and consistency across all areas (64). On the other hand, the Kimball approach emphasizes dimensional modeling to support analytical performance and usability for decision support (66). The Data Vault model further broadens this classical paradigm by creating agile, auditable, and historical structures to support regulatory compliance and incremental evolution–things that are particularly needed in a busy, heterogeneous healthcare data environment. By comparison, emerging architectures such as the medallion architecture and the data lakehouse enforce schema layout and layer data quality functions at scale in scalable big data ecosystems. These approaches make it possible for unified analytics across structured as well as unstructured data sources (24). However, more decentralized paradigms, including data fabric and so forth, emphasize cross-organizational data sharing and independent multi-document governance enabled by metadata, a concern made more and more concrete for multi-institutional healthcare research done today (70). In healthcare, optimal DW design typically has a hybrid aspect, combining features from multiple paradigms in order to achieve interoperability, compliance and analytical performance across the evolving clinical ecosystems.

### 8.3 Data modeling techniques

To ensure queries perform well and have a high degree of flexibility in a modern DW, it should make effective data models. Many people still use the dimensional modeling method. Its focus is on both the star schema and snowflake schema. These models consist of fact tables connected to multiple dimension tables, enabling OLAP queries to efficiently perform multidimensional analysis through rapid slicing and dicing of data. The star schema features denormalized dimension tables, enhancing query speed at the cost of data redundancy, while the snowflake schema normalizes dimensions to reduce redundancy but may introduce more complex joins (60, 66). Trade-offs between normalization and denormalization carry with them significant implications for scalability, performance and maintenance. Denormalized schemas make query execution faster, but will complicate ETL/ELT processes and require more storage; normalized schemas facilitate updates and consistency yet introduce the danger of slower query response times (67). Hence schema evolution and flexibility are essential capabilities in modern DWs where one is likely to meet frequent business changes requiring schema design changes or add-ons. Modern table formats enable dynamic schema changes without any outage. The formats have features such as decentralized metadata management, snapshot isolation, compactions happening atomically, and hidden parts, which permit variance in the schema while ensuring that performance will not be compromised and proper compatibility is still guaranteed for older queries (67, 71). These capabilities allow you to process real-time data and compensate for the fact that query optimization in environments with many partitions using standard planning algorithms is too slow (72).

### 8.4 Data integration and ETL/ELT processes

DW construction is a fundamental process of data integration primarily completed through ETL/ELT tools. Recent developments, such as automated ETL/ELT pipelines optimized for metadata management and AI integration, have transformed this landscape. These modern pipelines are designed to reduce manual workload for data engineers, enhance reliability, adapt dynamically to schema changes, address data quality issues, and ensure timely data availability (73). Nonetheless, even with technological advances as the backdrop ETL/ELT still presents problems relating to crisis management of data quality, coping with changes in data schema and interconnecting different data sources (74).

Maturity models for ETL/ELT processes have been developed to structure improvements from an *ad hoc* to a better-organized mass-movement technology. These models consist of Key Process Areas (KPAs) and Quality Objectives (QOs) that guide organizations in implementing structured, repeatable, and quality-assured ETL processes (74). However, new technologies such as ZeroETL paradigms and Incremental View Maintenance aim to reduce pipeline complexity and performance latency by enabling continuous, high-throughput upstream data processing within DWs. This pattern reduces the reliance on external stream processing engines and increases the support level for complex, high-concurrency workloads. Closer cooperation with cloud platforms and serverless architectures further reduces batch processing cycle times and cost (72).

### 8.5 Metadata and schema management

Efficient metadata management and schema are essential for coping with the complexity of modern DWs. Decentralized metadata management strategies have emerged to address the centralized bottlenecks characteristic of traditional systems. Apache Iceberg, for its part, makes use of distributed metadata management in conjunction with snapshot isolation and atomic commit support to provide consistent views of data while also allowing concurrent schema updates (71). This capability makes dynamic management of schema practical, therefore promoting greater business agility by reducing downtime and eliminating manual input. Integrating metadata into governance frameworks is vital for securing data lineage, assuring data quality and meeting regulatory commitments. More advanced governance constructs embody AI lifecycle stages within metadata management processes and enable metadata compliance (75). Automated governance processes improve auditability, reduce operational overhead, and align governance activities with privacy policies and compliance standards such as GDPR, Basel III, and CCPA. Schema evolution strategies focus on minimizing impact during schema changes, supporting zero-downtime operations. These include version-control mechanisms that ensure backward compatibility and allow consumers to query data seamlessly under different schema versions (71). Still, a major interoperability challenge arises when systems must integrate across large-scale multi-vendor data ecosystems using a variety of different metadata and schema management tools (75).

### 8.6 Data governance and quality management

Data governance frameworks ensure the accuracy, consistency, and availability of data assets critical for trustworthy analytics. Automated quality monitoring systems tailored for analytic use cases continuously assess data integrity, completeness, and freshness, thereby supporting reliable decision-making (75). Governance activities must align closely with business objectives and compliance mandates to maintain organizational trust and regulatory adherence (76). The integration of metadata and lineage systems provides comprehensive visibility over data flows and transformations, improving auditability and enabling regulatory reporting (75). Successful governance relies on a blend of organizational readiness, cultural alignment, and technical capabilities.

### 8.7 Advanced analytics and AI/ML integration

Modern DWs integrate AI and ML pipelines to generate deeper analytical value, serving as feature engineering centers and operational data foundations that support end-to-end ML processes, including training, deployment, and monitoring (68). Despite the advantages, integrating ML workflows into existing warehouse systems poses challenges, including managing model production environments, data lineage, and operational consistency (77). Real-time architectures incorporating streaming ETL/ELT and incremental view maintenance support low-latency analytics required for predictive modeling and business-critical applications (67). Applications span industries such as healthcare, where predictive analytics drive hospital resource optimization and patient outcome (49). Future trends highlight the growing adoption of cloud-native AI governance frameworks that facilitate automated compliance and continuous improvement (75). However, scalability challenges remain for managing AI workloads at enterprise scale, requiring innovations in autonomous ETL/ELT and data quality monitoring processes (73).

So far, the principles of architecture and methods have been discussed. Now they will be given life in a concrete setting. The following section describes the application of these concepts in actual practice, specifically the development of an IBDDW (inflammatory bowel disease DW).

## 9 The case study in IBD—BiotherDW

**Figure 2:**
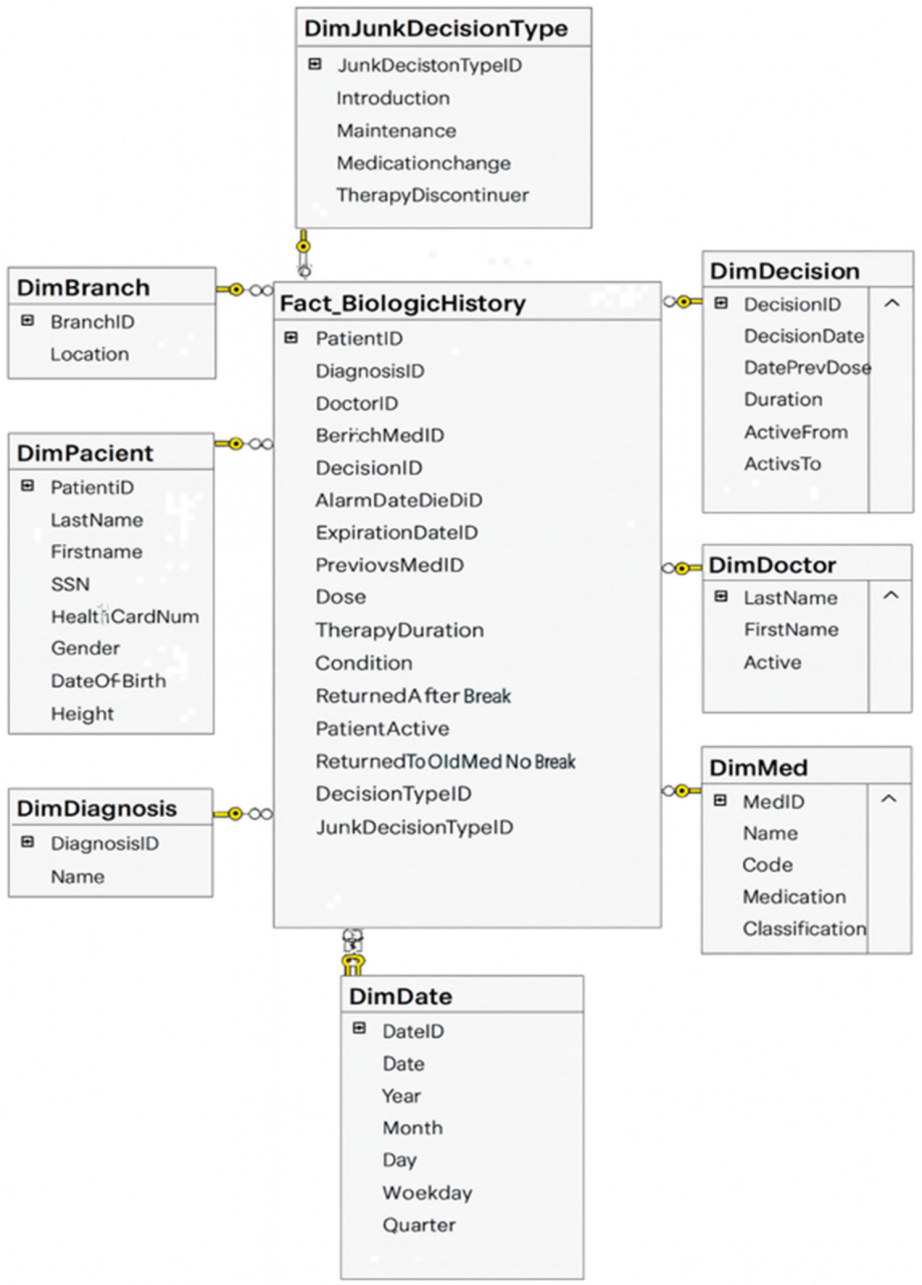
Excerpt of the BiotherDW schema.

Inflammatory bowel disease (IBD) exemplifies the complexity and chronic nature of modern diseases, requiring longitudinal, cross-disciplinary data integration. It thus serves as an insightful field test for applying DW architecture to real-world medical applications (23). The BiotherDW system, a national data infrastructure, primarily supports biologic therapy in Serbian IBD centers, demonstrating how fundamental DW principles can be effectively applied to deliver real-time clinical benefits as well as long-term research value (25, 65). The Biother team chooses a DW as a foundational layer in a phased modernization plan. Experts first stabilize governance and structure through a DW, then gradually extend toward a lakehouse architecture—integrating curated DW data with raw and semi-structured data in the lake (24, 31). The data architecture involves designing a modern DW using medallion architecture. Metadata-driven ETL/ELT frameworks are responsible for extracting data from source systems, transforming it to meet quality standards, and loading it into the DW. Data modeling emphasizes creating fact and dimension tables optimized for analytical query performance (66, 73). An excerpt of the BiotherDW schema is shown in Figure 2.

### 9.1 Metadata driven ETL/ELT

The existing BiotherDW solution has been developed based on many years of experience and a deep understanding of the principles of Kimball's and Inmon's methodologies, combining their best practices into a unique, customized medallion architecture (24, 64, 66). The ETL/ELT development is carefully metadata-driven, ensuring high automation, flexibility, and precision in data processing. This approach should guarantee scalability, standardization, and optimal management of the complex BI environment (73, 75). In developing this system, a strategy was used that combines a stable, denormalized model with full metadata control, integration with master data, and separation of views from physical tables with potentially flexible analytical base tables for analytics and AI. Master data, such as patient, is organized and managed at a higher level of aggregation to facilitate easier and consistent data management across the entire BI system. Such an approach ensures more effective data governance, reduces duplication, and promotes integrity by maintaining master data coherently at both detailed and aggregated consolidation levels.

To support real-time queries and audit trails, BiotherDW architecture emphasizes auditability as a key feature. The AuditID framework enables operational monitoring, such as performance diagnostics and pipeline health checks. In modern DW, this framework plays a central role in ensuring full traceability and auditability of data. It serves as a unique identifier, linking each warehouse record to the exact ETL/ELT process execution that created or modified it. This connection allows organizations to trace data lineage, knowing exactly when, how, and by which job each record entered the system. In practice, AuditID functions as a foreign key that connects fact and dimension tables to an audit log table, which contains detailed metadata about ETL/ELT executions. This metadata typically includes the job name, source system, start and end times, number of rows processed, and load status.

### 9.2 Medallion architecture

**Figure 3:**
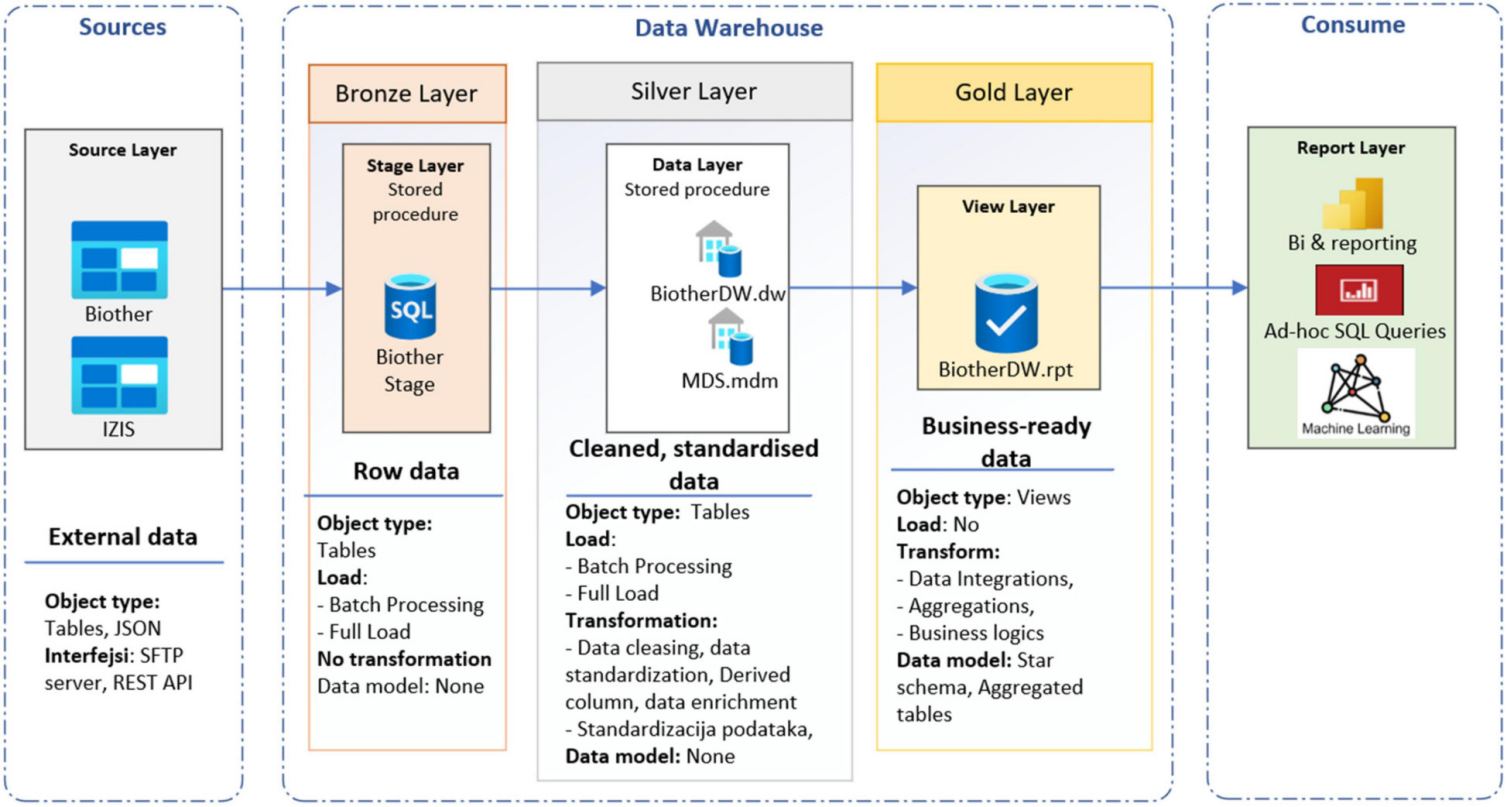
Medallion architecture of BiotherDW.

Medallion architecture (Figure 3) is a data organization and processing framework primarily used in modern data lakehouse environments (24, 68). The medallion pattern facilitates metadata-driven ETL/ELT processes, modular pipeline development, and gradual data validation, which together ensure data accuracy and seamless support for both traditional BI and AI workloads. The DW is the destination: a stable, curated analytical repository. The medallion architecture is the journey: a pipeline framework that progressively transforms data into trusted repository. Medallion architecture is a multi-layered data design pattern that organizes and progressively refines data as it flows through distinct stages called bronze, silver, and gold layers (24, 31). This structured approach enables scalable data management, improved data quality, and efficient analytics. The bronze layer represents the landing zone for raw, unprocessed data as it is ingested from various sources. The silver layer contains cleaned, deduplicated, and conformed data, enriched with metadata and standardized into a consistent schema. The gold layer consists of highly refined, aggregated, and business-ready data arranged for optimal query performance and integration into dashboards, reports, and decision-making workflows. The RPT schema is used as a view layer. This tiered architecture balances flexibility with governance by separating raw data ingestion from curated datasets, thus improving traceability, reliability, and operational efficiency (67).

In healthcare, medallion architecture offers a clear lineage and trust framework for clinical data pipelines. BiotherDW consolidates data from various clinical and systemic operational systems, integrating EHRs (diagnoses; prescriptions), laboratory platforms (fecal calprotectin; CRP), radiology archives (MRE; ultrasound), histopathology, and national registries. The BiotherDW system primarily sources data from two databases: the Biother transactional database (Biother) and the Serbian patient database (IZIS, Integrated Healthcare Information System). Biother is a software solution designed to manage patients undergoing biological therapy, providing numerous benefits. These include digital form submission and tracking for each patient, access to their health history, scheduling colonoscopy appointments, daily therapy planning and monitoring, and automatic generation of monthly medical decisions and reports for commission approval. It also helps forecast medication needs more accurately each month. IZIS is the central electronic health system that manages comprehensive medical and health data of patients, healthcare professionals, institutions, medical interventions, electronic referrals and prescriptions, as well as appointment scheduling and diagnostics. It offers unified patient data management across the entire healthcare system, enhancing efficiency, quality, and access to services through digitalization and interoperability among public, private, and military health institutions.

The current core BiotherDW includes machine learning and AI components, though their use remains limited and mainly applied to specific analytical tasks rather than large-scale operations. However, it has been designed from the beginning to support AI. Moving forward, developing federated learning capabilities that allow collaborative model training among distributed BiotherDW nodes without transferring raw data. Our current infrastructure features modules like dimensionality reduction and interactive binning for feature engineering, using Python-based preprocessing.

## 10 Conclusion

Healthcare organizations need a data repository to integrate fragmented clinical, administrative, and operational data into a single, consistent source of truth that supports reliable reporting and decision-making. It enables data quality, traceability, and regulatory compliance, providing a trusted foundation for analytics, research, and performance monitoring across the healthcare system. Most healthcare organizations continue to use a well-structured DW as the core of their analytical infrastructure, while data lakes and lakehouses serve mainly as complementary environments for large-scale learning and exploration. The DW remains the most trusted layer for ensuring data accuracy, lineage auditing, and clinical consistency, even as newer architectures evolve toward greater standardization. Thus, rather than replacing the DW, lakes and lakehouses extend its role within modern hybrid ecosystems that combine historical data management with cloud-based analytics and big-data workflows.

Stakeholders in healthcare data include patients, clinicians, researchers, administrators, policymakers, and technology providers, each relying on data to inform decisions, improve outcomes, and optimize operations. They benefit through enhanced care quality, evidence-based research, operational efficiency, and policy planning, enabled by secure, well-governed data sharing and advanced analytics within healthcare data infrastructures.

The complexity of healthcare DWs arises from multiple interrelated challenges, including data integration across heterogeneous sources, ensuring data quality amid bias and imbalance, safeguarding security and privacy, and maintaining scalable performance in resource-constrained environments. Technical skill shortages, regulatory constraints, interoperability barriers, and digital transformation pressures further complicate effective DW deployment. The success of healthcare DWs increasingly depends on interdisciplinary collaboration, continual workforce development, and adaptive governance frameworks. The construction of a modern DW is a multifaceted endeavor requiring the integration of scalable architectures, flexible data modeling techniques, robust ETL/ELT pipelines, and comprehensive governance frameworks. Security considerations, real-time processing capabilities, and AI/ML integration are critical to meeting contemporary business demands and regulatory landscapes. Emerging paradigms such as data lakehouses offer promising directions for next-generation data platforms, addressing the limitations of traditional centralized models and enhancing agility and scalability. Despite advances, notable challenges persist, including the need for greater automation in ETL/ELT and data quality management, scalability constraints in AI-driven governance frameworks, and overcoming cultural and organizational obstacles associated with new data platform adoption. Practitioners and researchers are encouraged to prioritize cloud-native architectures with adaptable data models, invest in metadata-driven governance frameworks, and leverage real-time data processing engines to maximize business agility and analytical effectiveness. By addressing these key components thoughtfully, organizations can build resilient, high-performance modern DWs that not only meet current analytical needs but also evolve with future technological and business transformations.

In diseases like IBD, a DW architecture is exemplified by the BiotherDW implementation, as reported. By combining ETL/ELT modularity and medallion architecture with the flexibility of a semantic schema that can adapt to various needs, along with regulatory-grade auditability and built-in privacy tools like synthetic data and federated learning, this system has become a modern blueprint for clinical platforms based on health data.
